# Choice models in Nordic long-term care: care managers' experiences of privilege and disadvantage among older adults

**DOI:** 10.1007/s10433-022-00697-z

**Published:** 2022-05-06

**Authors:** Sara Erlandsson, Helene Brodin, Lea Graff, Olli Karsio

**Affiliations:** 1 Department of Social Work, Stockholm University, Stockholm, Sweden; 2 The Danish Centre for Social Science Research, Copenhagen, Denmark; 3 Department of Social Sciences and Philosophy, University of Jyväskylä, Jyväskylä, Finland

**Keywords:** Nordic welfare state, Eldercare, Marketisation, Care managers, User choice, Social inequality

## Abstract

Consumer choice models have been introduced in eldercare services in several Western welfare societies. Choice models in eldercare emphasise the importance of individuals’ abilities to make informed choices and therefore entail a risk for increased inequalities among older adults with care needs. In the Nordic countries, such inequality risks are in stark contrast to universal policy ambitions of equal access to care services. Care managers, who are responsible for needs assessment for eldercare services, have a central role in implementing policies and, thus, have first-hand experience of their impact on older adults’ access to care. The aim of this study was to explore care managers’ experiences of how user choice affects older adults’ access to care services in three Nordic cities: Copenhagen, Tampere, and Stockholm. These cities were purposely selected as forerunners in marketisation, with different ways of implementing choice models. Semi-structured interviews with care managers were conducted in Copenhagen, Tampere, and Stockholm and analysed thematically. The findings indicate there are difficulties related to older adults’ ability to access information needed to make informed choices, as well as limitations in choice related to available services and personal finances. Further, care managers find that older adults’ abilities to overcome these difficulties are shaped by their health, education, language skills, and assistance from relatives. In order to reduce the risk of choice models increasing the gap between older adults with different resources and capabilities, there is a need to develop accessible information, as well as models for professional guidance.

## Introduction

Choice models have reorganised the provision of long-term care (LTC) across all Western societies (Burchart et al. 2015). While choice models are promoted by promises of empowerment and personalisation, they also entail risks of increased inequality among older adults because the skills and opportunities required to make informed choices are not equally distributed (Glendinning 2008; Meinow et al. 2011; Moberg 2017; Trætteberg and Fladmoe 2020).

This paper draws on Martha Alberston Fineman’s (2004, 2010, 2017) vulnerability theory in order to examine how choice models in Nordic LTC affect older adult’s access to care services. Specifically, it explores how care managers, the professional group responsible for needs assessment and allocation of care services, experience choice models in three Nordic cities: Stockholm, Copenhagen, and Tampere. These cities have all been forerunners of introducing choice models in LTC in Sweden, Denmark, and Finland, respectively, but have implemented choice in different ways (Szebehely and Meagher 2013). Given that Nordic LTC is funded, organised, and needs assessed at the local level, analyses of how care managers perceive the local operations of choice models can shed light on how system design features of choice models affect the universal policy ambition of the Nordic welfare states’ to ensure all older adults are provided equal access to publicly funded care services (Szebehely and Meagher 2018).

In this paper, LTC refers to both residential care and home care. The following sections present previous research on choice models and inequality, choice models in the context of Nordic LTC and how choice models are implemented in Copenhagen, Tampere and Stockholm. Thereafter, the analytical framework and methods are described, before the findings are presented and discussed.

## Choice models and (in)equality in long-term care

Research has shown that choice models in LTC tend to increase the significance of individual resources (Rodrigues and Glendinning 2015). Highly educated and native-speaking users have an advantage over less educated and non-native speaking users in terms of finding and processing information about different choices (Baxter et al. 2008; Baxter et al. 2020; Brodin 2018; Trætteberg and Fladmoe 2020). Poor health and high care needs may also limit one’s ability to access and process information, and many users therefore rely on family in choice situations (Baxter et al. 2020; Glendinning 2008; Meinow et al. 2011; Rodrigues and Glendinning, 2015; Vamstad 2016).

Research has also pointed out that choice models have the potential to ameliorate existing social inequalities among older adults through design features such as personalised information available in different languages and formats, as well as professional guidance (Baxter et al. 2008). However, studies suggest that choice models more often tend to have design flaws that increase inequalities, such as generic information presented in a uniform way without adaptions that account for impairments or foreign languages (Baxter et al. 2020; Dunér and Gustafsson 2020). Additionally, information about selectable providers is seldom presented in comparable ways and often lacks quality aspects, which also increases the importance of individuals’ abilities to find and evaluate information (Moberg et al. 2016; Rodrigues and Glendinning 2015).

## Choice models in the Nordic context

Choice models, under which users choose provider of services, were introduced in Denmark, Finland and Sweden during the 2000s. However, LTC is still publicly funded and what type and amount of services a person is entitled to is still determined through needs assessment conducted by care managers (Anttonen and Meagher 2013; Moberg 2017). In all three countries, choice models have been accompanied by tax rebates on both household and personal services that can be used instead of, or in addition to, needs-assessed services (Szebehely and Meagher 2018). Concurrently, all countries have witnessed an increased demand for LTC due to population ageing, as well as cutbacks in financing, resulting in stricter eligibility criteria for services and declining service coverage. Consequently, publicly funded care now focuses on the frailest individuals, and bodily help is prioritised over social needs and practical help (Kröger and Leinonen 2012; Olaison 2017; Rostgaard and Matthiessen 2019).

The interplay between choice models and cutbacks has led to major changes in Nordic LTC. Older adults who enter LTC have poorer health than before (Rostgaard et al. 2022). Simultaneously, access to care services has become more complex due to choices between care providers and/or the purchase of supplementary services, and the importance of both individuals’ abilities to make informed choices and financial resources has increased (Puthenparambil and Kröger 2016). Despite this, the legislation regarding the formal right to publicly funded care has not changed, and the universal policy ambition of equal access to care services remains (Ranci and Pavolini 2015).

This has created tensions and contradictions in the LTC sector that care managers, who implement both national and local policies in their daily work, deal with on a daily basis (cf. Brodkin 2011). Accordingly, care managers have first-hand experience of how locally implemented choice models unfold among various groups of older people. Through their accounts, this study explores how the design of choice models interacts with other system design features of Nordic LTC. It thereby seeks to add an account to the growing literature on inequality implications of choice reforms.

The aim of this paper is to explore care managers’ experiences of how choice models affect older adults’ access to care services: What are the difficulties and benefits of choice models? How are the difficulties and benefits related to the design of choice models and other design features of LTC? What are the implications for inequality?

## Choice models in Stockholm, Copenhagen, and Tampere

At the time of the study, Stockholm, Copenhagen, and Tampere had chosen different ways to implement choice models in LTC.

Stockholm had introduced a choice model for LTC which allowed service users to choose between public and private (for-profit or non-profit) providers in both home care and residential care. Distinctive for Stockholm was the large number of service providers. For example, a home care user could choose between more than 60 providers in each city district.

According to the law regulating choice models, the Act of System of Choice, local authorities have to appoint a provider, which could be public or private, for those who do not choose for themselves (Erlandsson et al 2013).

Copenhagen had introduced choice in both home care and residential care. However, in contrast to Stockholm, Copenhagen only had three service providers in home care (two private and one public). Users could also choose a ‘self-appointed carer’, who was employed by the municipality after an assessment of the carer’s qualifications. In Denmark, the Law on Free Choice of Provider of Practical Assistance and Personal Care has obliged local authorities to adopt choice models in home care since 2003, whereas it was optional in residential care (Bertelsen and Rostgaard 2013). There is no clear directive for how local authorities should act if an older adult does not want to choose provider, but according to interviews conducted in this study, older adults have to choose provider to receive services.

Tampere’s organisation of LTC differed from Copenhagen and Stockholm. Tampere used a service-integrator model called Kotitori that provided a single-entry point to privately and publicly paid home care services. Kotitori was publicly funded, but managed by a private for-profit company. This meant that care managers were either employed by a private company, or by the municipality. Care managers employed by Kotitori assessed the needs of older adults, and thereafter assembled service packages. The services were either privately paid out of pocket, or partly covered by local authorities if eligibility criteria were met (Tynkkynen et al. 2012). The mandate to decide on entitlement to publicly funded LTC lied with municipal care managers. Thus, Kotitori care managers cooperated with municipal care managers on decisions on eligibility. Needs-assed home care services were provided by public or private (non-profit or for-profit) providers appointed by a care manager (Karsio and Tynkkynen 2017). In residential care, Tampere had adopted a service voucher, which allowed service users with a solid economy to choose from a list of private providers as an alternative to public facilities. In Finland, service vouchers are regulated through the Act on Voucher System in Social and Health Care. If an individual does not qualify for, or turns down, the voucher, local authorities are required to arrange the services through a public or private provider (Karsio and Anttonen 2013).

A difference between the cities was that Copenhagen and Stockholm had introduced a choice model for both home care and residential care, whereas the service voucher in Tampere only covered residential care. In home care, service users could choose to either buy private services out of pocket or, if eligible, use publicly funded needs-assessed services. The service-integrator model meant that Kotitori care managers were directly involved in older adults’ purchases of privately paid services. On the contrary, in Copenhagen and Stockholm, purchases of privately paid services were made directly from providers and care managers had no part in them (Erlandsson et al. 2013; Karsio and Tynkkynen 2017; Bertelsen and Rostgaard 2013).

An additional difference between the cities regarded user fees for needs-assessed services in each country. In Denmark, home care was free of charge whereas there were fees for rent and services in nursing homes. In Sweden, there were user fees related to income and amount of services for home care as well as residential care. In Finland, there were income-related user fees for all services, and the fees were significantly higher than in Denmark and Sweden. On an aggregated level, user fees covered about 5% of the costs for LTC in Denmark and Sweden, and 15 to 20% in Finland (Karsio and Anttonen 2013; Moberg 2017).

## Analytical framework

According to Fineman (2010), vulnerability connected to old age is an inevitable human condition, as our embodiment makes us vulnerable to injuries, illness, and disability. Experiences of vulnerability are shaped by individual variations in abilities and accumulated resources, as well as social structures and the ways public institutions provide material and social resources to protect individuals from harm (Gordon-Bouvier 2020).

As an institution designed to compensate for vulnerability connected to old age, public LTC has the potential to ameliorate the impact of physical and cognitive decline. However, institutions tend to operate in ways that privilege some and create disadvantage for others (Fineman 2010, 2017). This means that although public LTC as an institution has the potential to ameliorate inequality, it can also reproduce, or even produce, inequality between groups of older adults. To reduce inequality, institutions and their professional representatives have to consider the different resources and abilities people have instead of treating everybody the same, which tends to reproduce existing inequalities. Hence, in order to avoid reproducing inequality, institutions need to strive towards equality in outcomes rather than sameness of treatment (Fineman 2004, 2017).

We draw upon this approach to analyse how choice models operate in Copenhagen, Stockholm, and Tampere in relation to other system design features of Nordic LTC and if this interplay contributes to counteracting or recreating existing social inequalities among older adults. Hence, we explore how choice models privilege or disadvantage different groups of older adults in relation to access to LTC in the context of Nordic policy. By using this approach, we strive to visualise problems related to failures in the organisation and operations of choice models in Nordic LTC instead of analysing individuals’ abilities (or lack thereof) to make informed choices (cf. Fineman 2010).

## Methods

This study used a qualitative comparative approach to explore similarities and differences between care managers’ experiences of differing implementations of choice models (cf. Hantrais 1999). The research team collaborated on the study design, sampling strategies, interview guide design, and data analysis, but the interviews were conducted by the researchers representing each city. (Stockholm was represented by two researchers.)

### Participants

A purposive sampling strategy that strived for variation in participants’ experiences was employed. Hence, participants with different lengths of work experience and occupational backgrounds and who were working in districts with different socio-demographic characteristics were recruited via enquiries to local authorities and Kotitori.

Ten care managers were interviewed in each city. Their professional experience ranged from 1 to over 30 years, and they had occupational backgrounds in social work, nursing, or occupational therapy. The care managers in Copenhagen were based in two city districts, one affluent and one more diverse, whereas those in Stockholm represented seven different districts. In Copenhagen and Stockholm, all participants were municipal employees. In Tampere, three participants were employed by Kotitori, and seven were municipal employees from different districts. All participants conducted needs assessment and informed older adults on LTC and available choices on a daily basis.

### Data collection

Semi-structured interviews were conducted using an interview guide covering six themes: work role in relation to choice models, needs of users, barriers to care, relationship with users, choice policies, and local implementation of choice. The interviews were conducted as dialogues in which the interviewees spoke freely about each topic, with as few interruptions as possible. According to participants’ wishes, the interviews were conducted at their workplaces. The length of the interviews was approximately one hour, and all were audio recorded. All participants received written information about the aim of the study as well as research ethical principles before consenting to the interview.

### Data analysis

The analysis was based on the six phases of thematic analysis suggested by Braun and Clarke (2006). Initially, interviews were transcribed verbatim in the original language. In addition, a detailed English summary of each interview enabled all authors to familiarise themselves with data in different languages. The summaries consisted of an overview of each theme, as well as relevant quotes translated into English. In phase 2, all authors agreed on a common coding frame and coded the full transcripts in their original language. All authors made a detailed English summary of the codes. We then compared our coding and made revisions when needed. In phase 3, the first author used the summaries of the coding to find potential common themes. In phase 4, each author used the full transcripts in their original language to review and validate the themes. In phase 5, we collectively refined the themes and merged them into three main themes: *Access to information*, *Limitations in choice*, and *Benefits of choice models*. In phase 6, the first author wrote a report on the themes to which all authors agreed. We then used the analytical framework to generalise the implications of choice reforms.

### Methodological considerations

A challenge in qualitative comparative studies is developing a framework that provides enough structure to ensure comparability while at the same time having the flexibility needed to generate in-depth data (Hantrais 1999; Quilgars et al 2009; Wendt 2020). Bearing this in mind, the interview guide consisted mainly of open-ended questions but also included follow-up questions that were asked unless the topics were spontaneously mentioned by the interviewees. This design ensured comparable data while at the same time allowing for adaptions, such as rephrasing or changing the order of questions, to the specific situation in each interview. Another challenge in the present study was language differences. Since interviews were conducted in different languages, it was not possible for the authors to access the full transcripts from all interviews. To establish trustworthiness, we worked iteratively with original transcripts and English summaries. For example, each author coded the full transcripts for their city and then made an extensive English summary to share with the team. The team then worked collectively, after which each researcher again validated the analysis using the full transcripts in the original language. In addition, to ensure that the meanings of words and expressions were not distorted during translation, the whole team collectively discussed not only what themes were manifested in each interview but also how they were manifested (cf. Gómez and Kuronen 2011; Wendt 2020).

## Findings

Even though choice models are implemented in different ways in each city, the findings point to many similarities between the cities. Therefore, findings are presented thematically, and differences between cities are discussed within each theme. Quotes have been translated to English.

### Access to information

A prominent topic in care managers’ accounts of their experiences of choice models in LTC was the importance of access to information about available choices and older adults’ ability to process information.

#### Information about available choices

When meeting older adults, care managers inform them about various aspects of LTC: eligibility criteria, fees, services, and options. Care managers perceived that the information could be overwhelming for the older adults. One care manager in Copenhagen said: ‘The sheer amount of information is tiring, and they can get so confused. They can hardly understand what services I am trying to grant them.’ Although care managers stated that older adults received a lot of information in both oral and written format, they problematised that the information was mostly general and not tailored to the older adult’s specific situation or needs. In many cases, detailed information about certain providers and services required internet use, which was perceived as problematic for older adults who did not have their own computers or internet access. This was illustrated by a care manager in Stockholm: ‘One can only find the providers on the internet, and the older persons often don’t have internet’.

Care managers in all cities reported concerns about older adults struggling to find information, however, those located in Stockholm described a specific challenge related to the large number of home care providers. One care manager confessed that she herself did not know the number of providers: ‘I have never really known how many providers we have had. It’s like a jungle.’

Finding information was perceived as a general problem for older adults, but care managers experienced that older adults had different opportunities to overcome this problem. Having younger relatives to assist in information seeking was perceived as an advantage. One care manager in Tampere illustrated: ‘Close relatives are the ones we work with. We offer information mainly to the close relatives.’

In addition, care managers perceived that older adults and relatives of certain backgrounds had fewer difficulties to navigate in LTC. The group with fewer difficulties was often referred to as ‘resourceful’ in the sense of being both educated and having financial resources. One Copenhagen care manager elaborated on the ‘resourceful’ group: ‘Many of them have checked out their options beforehand. Also, they often have relatives who can support them. They are used to seeking things out.’

In contrast to the ‘resourceful’ group, care managers portrayed older migrants, especially those who have immigrated late in life, as a disadvantaged group. Care managers perceived that it is harder for a person to understand how to access LTC if they lack general knowledge of welfare services. Another difficulty was the lack of information in different languages. One care manager in Copenhagen explained: ‘When you consider how hard it is for anybody to navigate the system, if you don’t understand what people tell you, it gets really, really difficult!’.

The disadvantages for older migrants were mentioned by care managers in all three cities but most so in Stockholm and least in Tampere. One probable explanation for this is that Stockholm is the city with the highest proportion of foreign-born older adults, and Tampere is the city with the lowest.

#### Ability to process information

The care managers provided many examples of older adults struggling not only to find information but also to make sense of information. A common experience was that the choice process causes a lot of stress and that many older adults are too ill to have the energy needed to make an informed decision. A care manager in Copenhagen stated: ‘We are dealing with ill people or very ill people. For them, it is important to get the help they need and get their life back on track. For them, the freedom to have the choice might not be as important, and they might not even have the energy to think about it.’ The difficulties experienced by these older adults were enhanced by the fact that choices often have to be made under less-than-ideal circumstances, for instance, in situations of acute illness. A care manager in Stockholm illustrated: ‘The person has been through a rough time—perhaps they almost died. And then we arrive, and we give them all the information in one hour—about the services, about the application process, and about the choice of provider. The person has poor hearing and sees poorly, and their three children are fighting…’.

One aspect that was repeated in virtually all interviews was the difficulty of discussing choice with older adults with dementia. In some cases, the affected person has no awareness of their illness and does not want any services at all. Under these circumstances, the care managers focused on convincing the person to accept services rather than covering all the aspects of the information. If possible, they cooperated with relatives, who then made choices instead of the older adult, although they were unsure whether this was a legal practice. One care manager in Tampere said: ‘Who makes the choice for them when they cannot make it themselves? The customer has to understand what they are doing and what they are committing to. Is it right if their next-of-kin makes the choice?’.

### Limitations in choice

The second theme addresses limitations in choice regarding available services and personal finances.

#### Available services

Care managers stated that they could not always grant services adjusted to a person’s individual needs. Care managers in all cities accounted for a standardisation of services, leading to available services being either practical (help with the home) or personal (help with the body). As a consequence, there was a general lack of services addressing social needs, as one care manager in Copenhagen illustrated: ‘There are those who have needs related to loneliness or anxiety, and it’s really hard to support them with that. We have no services aimed at these needs.’

The standardisation of services entails difficulties in meeting complex needs, such as mental health problems and substance abuse in combination with physical problems or dementia. In all three cities, care managers stated that it is hard to meet needs that do not match the available services. A care manager in Tampere concluded: ‘I am quite helpless because I have no services to offer them.’

When it comes to having services granted, relatives can be of importance. Care managers reported that older adults who have relatives who know what services there are and what arguments to use in order to get these services are in a better position to receive services since these relatives tend to act as advocates for their next-of-kin and argue their case for them. This is especially so if the older adults are on the verge of being eligible for a service, as illustrated by a care manager in Copenhagen: ‘Sometimes, you might give in a little, and it might be a little easier to grant the service than if nobody is pushing for it.’ In line with this, care managers expressed concerns that older adults who do not have relatives to inform them might miss out on services because they are unaware of them.

#### Personal finances

Another aspect of limitations in choice regarded personal finances. This subject was most commonly raised by care managers in Tampere, where older adults with enough financial resources could access choices either through the private market for home care services or through the voucher system for residential care. One care manager explained the importance of personal finances: ‘What gives you options is money. If you receive a low pension and you only have a little bit of money at your disposal, then you don’t have that freedom of choice.’

However, the importance of personal finances extends beyond the choice model in Tampere, where care managers experienced that older adults cut back on services or even refrained from services due to high customer fees, even for public services. One care manager in Tampere illustrated: ‘Yesterday, I received a call from a customer who had just started receiving a service. The customer told me their monthly income, and I calculated what the monthly fee would be. The customer was terrified and had to cut back on the services.’

Care managers in Stockholm had also experienced users being reluctant to accept services due to user fees, although they did not report this to the same extent as the care managers in Tampere. In Stockholm, this was perceived as most common among users with smaller care needs, who felt that the costs for services were disproportionally high. One care manager in Stockholm said: ‘There are some who think they cannot afford it, that their financial situation would become too strained. And they sometimes decline services.’ In Copenhagen, where there were no fees for home care services, the care managers did not report that financial resources affected access to care services.

### Benefits of choice models

Care managers rarely mentioned any positive aspects of choice models, and when they did, there was no consistency between the cities. In Stockholm, care managers declared the emergence of providers with different language profiles to be an improvement for older migrants since the opportunity to receive care in one’s own language makes services more accessible. One care manager in Stockholm said: ‘Then they can understand the staff because they speak the same language. So, it is very beneficial that there are different providers of home care services who know different languages.’ However, other care managers problematised that this opportunity was only present for some of the largest language groups, and the providers usually made no guarantees that all staff could speak a specific language.

In Tampere, the city where older adults’ personal finances mattered the most, some of the care managers emphasised that the choice model benefited older adults with a good personal economy. However, they approached this fact from different perspectives. As shown in the section on personal finances, some expressed concern for those least well off. Others argued that it is only fair that people with more money can access more or better services. When discussing the voucher for residential care services, which is only available for the more well off, one care manager in Tampere stated: ‘Why should everybody have the same ‘shit’, so to speak? Why shouldn’t everyone who has money use it for their own well-being? I think it is a great addition.’

## Discussion

This paper has explored care managers’ experiences of how choice models affect older adults’ access to LTC in three Nordic cities. The findings suggest that choice models entail more difficulties than benefits. Across all cities, the findings revealed difficulties related to accessing information needed to make informed choices. These difficulties are related to the fact that the information older adults receive in relation to making choices is general, while older adults are expected to find more specific information needed to make informed choices by themselves. In line with earlier studies, the care managers in our study stressed that difficulties in accessing information are exacerbated when the older adult is in a poor state of health and has a lack of energy and/or reduced cognitive capacity (Glendinning 2008; Rodrigues and Glendinning, 2015; Vamstad 2016). This suggests that choice models increase the impact of vulnerability connected to old age (cf. Fineman 2010, 2017).

The findings also revealed limitations in choice. In all three cities, care managers reported that older adults’ opportunities to receive services adjusted to individual needs are restricted by the type and form of need. Available services are either practical (help with the home) or personal (help with the body), which means that people with complex needs and social needs, such as loneliness, are disadvantaged. However, these problems are more related to cutbacks and standardisation of services (Kröger and Leinonen 2012; Rostgaard and Matthiessen 2019) than they are direct consequences of choice models.

Taken together, these findings have implications for two different aspects of inequality in access to care: unequal opportunities to make informed choices and unequal opportunities to having ones needs met. This shows that sameness in treatment is not enough to achieve equality in outcomes (Fineman 2004, 2017). Even if all older people are treated the same in needs assessment and receive the same information about available choices, there will still be different outcomes because people have different needs and abilities.

The differences between the cities regarding the benefits of choice models and the importance of financial resources show that choice models may have different implications in different contexts. The large number of providers in Stockholm can be an obstacle in choice but also entails the benefit of providers with language profiles. Most variation between the cities is related to the importance of personal finances. In Tampere, the impact of personal income seems much more prominent than in the other cities. This can be attributed to high user fees in publicly funded Finnish LTC, in combination with a voucher system for residential care that is only accessible for those with a solid personal economy. Also, in Stockholm, the care managers experienced that older adults with fewer resources struggle with the user fees. In Denmark, there are no fees for home care services, and care managers in Copenhagen did not perceive personal finances as a barrier to care. The fact that care mangers experience that user fees restrict access to LTC in two of the three cities is worrying from an equality perspective, and deserves more attention in the future research.

Altogether, the findings indicate that the operations and organisations of choice models reproduce existing inequalities (cf. Fineman 2010; 2017). A significant design flaw demonstrated in this study regards the difficulties in finding information and the lack of tailored information. Although this is a problem for most older adults, it has inequality implications since people have different opportunities to overcome these difficulties (cf. Fineman 2010). The findings confirm that older adults with resources such as, higher education, or who have relatives with higher education, stand a better chance of finding information (Baxter et al. 2020; Trætteberg and Fladmoe 2020). Findings from this study also suggest that relatives can have an impact on the needs assessment process and, thus, on access to services. This result is tentative and needs further investigation, but if confirmed, it would suggest inequality in treatment.

In addition, the findings of this study point to the conclusion that general knowledge of the welfare system is an advantage, whereas newcomers to the system, such as older migrants, are disadvantaged. Although non-native speakers are generally identified as a disadvantaged group in research on choice models (cf. Baxter et al. 2008), the results from this study also suggest that the establishment of providers with language profiles is a possible benefit of choice models. However, this does not compensate for the lack of accessible information. Consequently, people with limited abilities to find and process information are clearly disadvantaged in the current organisation of choice models in Nordic LTC, whether due to physical illness, loss of cognitive capacity, or being non-native speakers (Baxter et al. 2008; Brodin 2018).

### Strengths and limitations

One limitation of this study is that we only present the perspectives of care managers and, thus, have no empirical data of older adults’ experiences. By interviewing older adults about their experiences, we could have gained insight into individual experiences of choice models. However, interviewing care managers, who meet many older adults in their work, provided an overview of how choice models affect access to services. Another limitation is that we rely solely on interview data reflecting the care managers’ experiences, which means we cannot determine actual differences in older adults’ access to LTC within, or between, the three cities. Despite these limitations, the study points to significant design flaws and contributes to the knowledge on inequality implications of the interplay between how choice models are implemented and general features of LTC.

### Policy and practice implications

In line with other studies, our findings suggest that older adults have different resources and abilities to overcome barriers raised by design flaws. Like Fineman (2010), we perceive inequality as inevitable in the sense that people have different abilities. However, system design is changeable, and it is possible to design a system that takes individual differences into account. In terms of policy, making the system more accessible to everyone could reduce the differences between those who can overcome barriers and those who cannot. The present organisation of LTC presumes healthy users who have the ability to act like customers and seek and evaluate information on services and providers. In contrast, care managers articulate that the people they meet are far from this ideal type.

The present study has identified general system design features that could potentially ameliorate inequalities among older adults: low user fees, services that match actual needs and not only the most basic ones, and more transparent choice models with tailored information. There is also a need for more targeted interventions, such as information in different formats, including easy-to-read formats; information in foreign languages; and professional guidance for those who do not have a network of relatives to rely on, as well as other vulnerable groups, such as non-native speakers and people with dementia (cf. Baxter et al. 2008; Dunér and Gustafsson 2020).

## Conclusions

The findings revealed difficulties related to design features of choice models but also difficulties that are not directly related to choice models. According to care managers, older adults with poor health, low education, poor language skills, and no close relatives are the most negatively affected by these difficulties. To prevent the gap from increasing between older adults with greater resources and those with fewer resources, measures are needed to make LTC more transparent and accessible for everyone.

## Data Availability

The data analysed in the current study cannot be made available due to confidentiality agreements between researchers and participants.
